# Laparoscopic versus open appendectomy: a retrospective cohort study assessing outcomes and cost-effectiveness

**DOI:** 10.1186/s13017-016-0102-5

**Published:** 2016-08-30

**Authors:** Antonio Biondi, Carla Di Stefano, Francesco Ferrara, Angelo Bellia, Marco Vacante, Luigi Piazza

**Affiliations:** 1 Department of Surgery, Vittorio Emanuele Hospital, University of Catania, Via Plebiscito, 628, 95124 Catania, Italy; 2 General and Emergency Surgery Department, Garibaldi Hospital, 95100 Catania, Italy; 3 Department of Medical and Pediatric Sciences, University of Catania, 95125 Catania, Italy

**Keywords:** Open appendectomy, Laparoscopic appendectomy, Hospital cost, Appendicitis

## Abstract

**Background:**

Appendectomy is the most common surgical procedure performed in emergency surgery. Because of lack of consensus about the most appropriate technique, appendectomy is still being performed by both open (OA) and laparoscopic (LA) methods. In this retrospective analysis, we aimed to compare the laparoscopic approach and the conventional technique in the treatment of acute appendicitis.

**Methods:**

Retrospectively collected data from 593 consecutive patients with acute appendicitis were studied. These comprised 310 patients who underwent conventional appendectomy and 283 patients treated laparoscopically. The two groups were compared for operative time, length of hospital stay, postoperative pain, complication rate, return to normal activity and cost.

**Results:**

Laparoscopic appendectomy was associated with a shorter hospital stay (2.7 ± 2.5 days in LA and 1.4 ± 0.6 days in OA), with a less need for analgesia and with a faster return to daily activities (11.5 ± 3.1 days in LA and 16.1 ± 3.3 in OA). Operative time was significantly shorter in the open group (31.36 ± 11.13 min in OA and 54.9 ± 14.2 in LA). Total number of complications was less in the LA group with a significantly lower incidence of wound infection (1.4 % vs 10.6 %, *P* <0.001). The total cost of treatment was higher by 150 € in the laparoscopic group.

**Conclusion:**

The laparoscopic approach is a safe and efficient operative procedure in appendectomy and it provides clinically beneficial advantages over open method (including shorter hospital stay, decreased need for postoperative analgesia, early food tolerance, earlier return to work, lower rate of wound infection) against only marginally higher hospital costs.

**Trial registration:**

NCT02867072 Registered 10 August 2016. Retrospectively registered.

## Background

Appendicitis is the most common cause of surgical abdomen in all age groups [1, 2]. Approximately 7–10 % of the general population develops acute appendicitis with the maximal incidence being in the second and third decades of life [3]. Open appendectomy has been the gold standard for treating patients with acute appendicitis for more than a century, but the efficiency and superiority of laparoscopic approach compared to the open technique is the subject of much debate nowadays [3–5]. There is evidence that minimal surgical trauma through laparoscopic approach resulted in significant shorter hospital stay, less postoperative pain, faster return to daily activities in several settings related with gastrointestinal surgery [6, 7]. However, several retrospective studies [3, 8–14], several randomized trials [15–20] and meta-analyses [21, 22] comparing laparoscopic with open appendectomy have provided conflicting results. Some of these studies have demonstrated better clinical outcomes with the laparoscopic approach [15–17, 20, 23], while other studies have shown marginal or no clinical benefits [18, 19, 24–26] and higher surgical costs [4, 19, 24, 25]. Bearing in mind that laparoscopic appendectomy, unlike other laparoscopic procedures [27], has not been found superior to open surgery for acute appendicitis, we designed the present study to determine any possible benefits of the laparoscopic approach. The aim of this study was to compare the clinical outcomes (hospital stay, operating time, postoperative complications, analgesia requirement, time to oral intake and to resume normal activity) and the hospital costs between open appendectomy and laparoscopic appendectomy.

## Methods

### Patients

A retrospective observational study of patients admitted to a single institution (Department of Emergency Surgery, Garibaldi Hospital-Catania) between January 2004 and July 2011 with the diagnosis of appendicitis was conducted. Pregnant women and patients with severe medical disease (hemodynamic instability, chronic medical or psychiatric illness, cirrhosis, coagulation disorders) requiring intensive care were excluded. The decision about the type of the operation was made according to the preference and experience of the surgical team on duty. We analyzed 593 patient that met the inclusion criteria and their clinical data and hospital costs. The patients were divided into two groups: open appendectomy (OA) group and laparoscopic appendectomy (LA) group. The collected clinical data included demographic data, co-morbidities, initial laboratory findings, operation time, intraoperative findings (acute, gangrenous or perforated appendix), time to soft diet, postoperative hospital stay, amount of analgesics and postoperative complications. We analyzed data on cost separately. The diagnosis was made clinically with history (right iliac fossa or periumbilical pain, nausea/vomiting), physical examination (tenderness or guarding in right iliac fossa). In patients where a clinical diagnosis could not be established, imaging studies such as abdominal ultrasound or CT were performed. Both groups of patients were given a prophylactic dose of third-generation cephalosporin and metronidazole at induction of the general anesthesia as part of the protocol. OA was performed through standard McBurney incision. After the incision, peritoneum was accessed and opened to deliver the appendix, which was removed in the usual manner. A standard 3-port technique was used for laparoscopic group. Pneumoperitoneum was produced by a continuous pressure of 12–14 mmHg of carbon dioxide *via* a Verres canula, positioned in infraumbilical site. The patient was placed in a Trendelenburg position, with a slight rotation to the left. The abdominal cavity was inspected in order to exclude other intrabdominal or pelvic pathology. After the mesoappendix was divided with bipolar forceps, the base of the appendix was secured with two legating loops, followed by dissection distal to the second loop. Then, the distal appendicular stump was closed to avoid the risk of enteric or purulent spillage. The specimen was placed in an endobag and was retrieved through a 10-mm infraumbilical port. All specimens were sent for histopathology. The patients were not given oral feed until they were fully recovered from anesthesia and had their bowel sounds returned when clear fluids were started. Soft diet was introduced when the patients tolerated the liquid diet and had passed flatus. Patients were discharged once they were able to take regular diet, afebrile, and had good pain control. The operative time (minutes) for both the procedures was counted from the skin incision to the last skin stitch applied. The length of hospital stay was determined as the number of nights spent at the hospital postoperatively. Wound infection was defined as redness or purulent or seropurulent discharge from the incision site. Seroma was defined as localized swelling without redness with ooze of clear fluid. Paralytic ileus was defined as failure of bowel sounds to return within 12 h postoperatively. The study protocol was received and approved by the Institutional Review Board and the Ethics Committee of Garibaldi Hospital. Waiver of informed consent from patients was approved because of the observational nature of the study. This study uses compliance with STROBE criteria, a checklist which has been developed to strengthen reporting standards in epidemiological research [26].

### Statistical analysis

Categorical data were presented as frequencies and percentage and compared by the Chi-square test. Parametric and nonparametric continuous data were presented as mean and standard deviation and evaluated by the Student’s *t* test and Mann–Whitney *U* test respectively. Comparisons between the two groups were made on an intention-to-treat basis. Thus, patients in the laparoscopic-assisted group converted to the open procedure were not excluded from the analysis. The sample size for our study was calculated based on an analysis of sample sizes required for each of the parameters (operative time, length of hospital stay, postoperative pain, complication rate, return to normal activity and cost) for an α = 0.05 and a power of 90 %. A P-value of 0.05 was considered as significant. All calculations were performed by using the SPSS software package version 17.0 (SPSS Inc., Chicago, IL).

## Results

Out of 593 patients with acute appendicitis, 310 patients underwent open appendectomy and 283 patients underwent laparoscopic appendectomy. Demographic data and preoperative clinical feature between OA group and LA group are showed in Table 1. There were no significant differences with respect to age and associated co-morbidities. On the contrary, the difference in gender and in the white blood cell count at presentation was statistically significant. Out of the total 310 open procedures, 214 (69 %) were performed for uncomplicated appendicitis and 96 (31 %) for complicated disease including appendiceal perforation with local or widespread peritonitis. In the laparoscopic group, 241 (85 %) procedures involved uncomplicated disease and 42 (15 %) complicated appendicitis. Noteworthy, we did not observe differences between groups for all the grades of appendicitis (Table 2). In our study, the mean ± standard deviation (SD) operative time of 54.9 ± 14.7 min for the LA group was longer than the mean operative time of 31.36 ± 11.43 min for open appendectomy (*P* <0.0001). The laparoscopic group required fewer doses of parenteral and oral analgesics in the operative and postoperative periods compared with the open appendectomy (*P* <0.0001). Bowel movements in the first postoperative day were observed in 93 % patients subjected to laparoscopic appendectomy and 69 % in the open group (*P* <0.001). As a result, 85 % patients in the laparoscopic group and 62 % in the open group were able to tolerate a liquid diet within the first 24 postoperative hours (*P* <0.001). Hospital stay was significantly shorter in the laparoscopic group with a mean ± SD of 1.4 ± 0.6 days compared with 2.7 ± 2.5 of the open appendectomy group (*P* = 0.015). A highly significant difference existed between the 2 groups in time taken to return to routine daily activities, which was less in the laparoscopic group with a mean 11.5 ± 3.1 days compared with mean 16.1 ± 3.3 days in the open appendectomy group (Table 3). We observed a greater overall incidence of complications in open surgery than in laparoscopic surgery. A total of 29 complications occurred in the laparoscopic group, while 55 complications occurred in the open appendectomy group, as summarized in Table 4. We did not observe a significant difference between groups in vomiting, paralytic ileus, intrabdominal abscesses and hemoperitoneum. Differences in wound infection and wound dehiscence were significant (*P* <0.001) (Table 4). Analysis of hospital costs are presented in Table 5. As regards laparoscopy, it is well known that the longer operative and anaesthesiological time are more expensive than the cost of the open approach (that uses reusable instruments and few and cheaper equipment). However, the shorter hospital stay (mean 1.4 ± 0.6 days) in the laparoscopic group kept low the ward cost in comparison to the open group. So, the total hospital cost for each patient of the LA group was only 150 € higher compared to patients in the OA group.

**Table 1:** Demographic and preoperative clinical data

	Open appendectomy (*n* = 310)	Laparoscopic appendectomy (*n* = 283)	*P*
Gender			<0.001
Male	184 (59.3)	121 (42.7)	
Female	126 (40.7)	162 (57.3)	
Mean age	29.66 ± 15.13	27.75 ± 14.24	0.57
WBC count (per mm^3^)	14903 ± 4686	13346 ± 5450	0.0002
Co-morbidities			0.244
CAD	6 (1.9)	5 (1.7)	
Hypertension	18 (5.8)	9 (3.1)	
COPD	9 (2.9)	6 (2.1)	
DM	12 (3.8)	5 (1.7)	

Data are number (%) or mean ± standard deviation values, as indicated. *WBC* White blood cell, *CAD* Coronary artery disease, *COPD* Chronic obstructive pulmonary disease, *DM* Diabetes mellitus

**Table 2:** Surgical findings

	Open appendectomy (*n* = 310)	Laparoscopic appendectomy (*n* = 283)	*P*
Surgical findings, n (%)			0.074
Uncomplicated acute appendicitis	214 (69.0)	241 (85.2)	
Gangrenous appendicitis	24 (7.7)	12 (4.2)	
Appendiceal abscess	38 (12.3)	22 (7.8)	
Peritonitis	34 (11.0)	8 (2.8)	

Data are number (%)

**Table 3:** Operative and postoperative clinical data

	Open appendectomy (*n* = 310)	Laparoscopic appendectomy (*n* = 283)	*P*-value
Operative time (min)	31.36 ± 11.43	54.9 ± 14.7	<0.0001
Bowel movements (1^st^ POD)	214 (69.0)	263 (92.9)	<0.001
Time until diet (1^st^ POD)	192 (61.9)	241 (85.2)	<0.001
Parenteral analgesics (doses/day)	1.5 ± 0.6	1.0 ± 0.5	0.001
Oral analgesics (doses/day)	2.00 ± 2.26	1.86 ± 1.14	<0.0001
Hospital Stay (day)	2.7 ± 2.5	1.4 ± 0.6	0.015
Return to normal activity (day)	16.1 ± 3.3	11.5 ± 3.1	<0.001

Data are number (%) or mean ± standard deviation values, as indicated. *POD* postoperative day

**Table 4:** Minor e major postoperative complications for open and laparoscopic appendectomy

Postoperative complications	Open appendectomy (*n* = 76)	Laparoscopic appendectomy (*n* = 29)	*P*
Minor			
Vomiting	17 (22.4)	13 (44.8)	0.621
Paralytic ileus	11 (14.5)	8 (27.6)	0.618
Wound infection	33 (43.4)	4 (13.8)	<0.001
Major			
Wound dehiscence	13 (17.1)	0 (0.0)	<0.001
Intra-abdominal abscess	1 (1.3)	4 (13.8)	0.147
Hemoperitoneum	1 (1.3)	0 (0.0)	0.339

Data are number (%)

**Table 5:** Analysis of hospital cost

	Laparoscopic appendectomy	Open appendectomy
Equipment cost	1245 €	50 €
Theatre cost	300 €	300 €
Ward cost	800 €/night	800 €/night
Anesthesia cost of mean operative time	350 €	280 €
Total cost of mean in-patient hospital stay	2965 €	2810 €

## Discussion

Acute appendicitis is the most common intra-abdominal condition requiring emergency surgery [25]. The possibility of appendicitis must be considered in any patient presenting with an acute abdomen, and a certain preoperative diagnosis is still a challenge [28, 29]. Although more than 20 years have elapsed since the introduction of laparoscopic appendectomy (performed in 1983 by Semm, a gynaecologist), open appendectomy is still the conventional technique. Some authors consider emergency laparoscopy as a promising tool for the treatment of abdominal emergencies able to decrease costs and invasiveness and maximize outcomes and patients’ comfort [30, 31]. Several studies [4, 10, 13, 16, 18, 32–34] have shown that laparoscopic appendectomy is safe and results in a faster return to normal activities with fewer wound complications. These findings have been challenged by other authors who observed no significant difference in the outcome between the two procedures, and moreover noted higher costs with laparoscopic appendectomy [3, 19, 20, 33, 35]. Anyway, a recent systematic review of meta-analyses of randomised controlled trials comparing laparoscopic versus open appendectomy concluded that both procedures are safe and effective for the treatment of acute appendicitis [36]. Total operative time in our series was significantly longer in the laparoscopic group than in open group (*P* <0.0001). Generally, the lack of experience of surgeons in the laparoscopic approach may contribute to a longer duration of the operation. By contrast, in the present study the learning curve effect was minimal as the surgeons performing the procedures were highly experienced in laparoscopic procedures, including laparoscopic bariatric surgery and colectomy surgery. So, in our series the longer operation time in laparoscopic appendectomy may be due to additional steps like setup of instruments, insufflation, making ports under vision and a phase of diagnostic laparoscopy. Length of hospital stay represents a critical factor that directly influences the economy and the well-being of the patient. We found that hospital stay was significantly shorter in laparoscopic group (*P* = 0.015) with a concomitant earlier bowel movements in patient managed laparoscopically, leading to earlier feeding and discharge from hospital. Our findings are in agreement with several studies that demonstrated a significantly short hospital stay for the laparoscopic approach [8, 22, 32, 33, 37]. In our Surgery Department, post-operative pain is assessed both subjectively *via* a visual analogue scale and objectively by the tabulation of analgesic use. In the present study, to prevent that the perception of pain may have been influenced by the patient’s enthusiasm for a novel technique, we used only the number of analgesics doses (oral and parenteral) required by individual patient to compare the 2 groups. In this series, parenteral and oral analgesic requirements were less in the laparoscopic group [parenteral 1 (mean); oral 1.86 (mean)] than in the open group [parenteral 1.5 (mean); oral 2 (mean)] and we found a statistically significant difference (*P* <0.001) in agreement with many other studies [15, 38, 39] that reported less pain in the laparoscopic group. Several studies showed no difference between open and laparoscopic appendectomy with respect to early return to activity and performance of daily activities. However, this issue is still debated because of the different definitions and classifications of “activity” in such studies [20, 40–43]. In this study we used the return to work as an endpoint with a mean time of 11.5 ± 3.1 days in the laparoscopic group and 16.1 ± 3.3 in the open group (*P* <0.001). Our results are in agreement with a study by Hellberg et al. [44] and other randomized clinical trials and meta-analysis.[4, 39] The mortality rate was nil in our study. The low mortality rates reported in previous research (0.05 % and 0.3 % rate in laparoscopic and open groups [4]) indicated that appendectomy, especially in absence of complicated disease, is a safe procedure regardless of the technique used [33]. In the present study, the overall complication rates were 24.5 % and 6.7 % for open and laparoscopic appendectomy respectively, with a rate of wound infection and dehiscence significantly higher in the open group (*P* <0.001). Wound infection is more common in complicated appendicitis and may not represent a serious complication *per se* but has a strong impact for convalescence time and quality of life of patients. In our study no statistically difference was observed in the intraoperative findings between the two groups (Table 2), so the lower rate of wound infection in laparoscopic group may be due to placement of the detached appendix into an endobag before its removal from the abdominal cavity, reducing contact with the fascial surfaces and minimizing contamination. Conversely, intra-abdominal abscess is a serious and life-threatening complication. We observed intra-abdominal abscess formation in 4 patients in laparoscopic group (4.1 %) and in 1 patient in the open group (0.32 %). These findings are consistent with other studies that showed an increased risk of intra-abdominal abscess after laparoscopic appendectomy compared with open surgery [32, 33]. Several hypotheses have been suggested to find possible explanations: mechanical spread of bacteria in the peritoneal cavity promoted by carbon dioxide insufflation, especially in case of ruptured appendix [25, 44–47], inadequate learning curve [32], the meticulous irrigation, instead of simple suctioning, of the infected area in severe peritonitis, that leads to contamination of the entire abdominal cavity, which is difficult to aspirate latter [35]. However, in our study this finding was not statistically significant (*P* = 0.147). The management of intrabdominal abscesses included percutaneous drainage as first-line therapy, and surgical procedures. Antibiotics were given before and after percutaneous drainage or surgery. Other observed postoperative complications included vomiting, paralytic ileus and hemoperitoneum (Table 4). The higher cost of laparoscopic instruments (1245 € in our Department) compared to the conventional technique (50 € in our Department) represents an obstacle to its greater use. However, because of the shorter hospital stay, the total cost for laparoscopic appendectomy (operating room + ward costs) was only 155 € higher than open appendectomy. In addition, Moore and al. demonstrated an economic benefit of laparoscopic appendectomy from a social perspective, since earlier return to daily activities is crucial, especially for patients who are young and lead a productive life [38]. Limitations of our study included the lack of evaluation of laparoscopic surgery in obese patients, as we did not collect data on body mass index (BMI). Moreover the follow up period was only limited to two weeks after hospital discharge.

## Conclusions

Our results showed the advantages of the laparoscopic approach over open appendectomy including shorter hospital stay, decreased need for postoperative analgesia, early food tolerance, earlier return to work, lower rate of wound infection, against only marginally higher hospital costs. Furthermore we found a considerable preference (during the collection of consent) of patients and a high satisfaction after the surgery in the laparoscopic group. Although the incidence of intra-abdominal abscess formation was higher after laparoscopic appendectomy, greater experience and improvements in our technique may have eradicated this catastrophic complication. Provided that surgical experience and equipment are available, laparoscopy could be considered safe and equally efficient compared to open technique and should be undertaken as the initial procedure of choice for most case of suspected appendicitis. However, since there is no consensus to the best approach, both procedures (open and laparoscopic appendectomy) are still being practiced actively deferring the choice to the preference of surgeon and patients. In the future, laparoscopic appendectomy could represent the standard treatment for patients with appendicitis and undiagnosed abdominal pain.

## Abbreviations

BMI: Body mass index
CAD: Coronary artery disease
COPD: Chronic obstructive pulmonary disease
CT: Computed tomography
DM: Diabetes mellitus
LA: Laparoscopic appendectomy
OA: Open appendectomy
POD: Postoperative day
WBC: White blood cell
